# Mean Species Abundance as a Measure of Ecotoxicological Risk

**DOI:** 10.1002/etc.4850

**Published:** 2020-09-16

**Authors:** Selwyn Hoeks, Mark A.J. Huijbregts, Mélanie Douziech, A. Jan Hendriks, Rik Oldenkamp

**Affiliations:** ^1^ Department of Environmental Science, Institute for Water and Wetland Research Radboud University Nijmegen Nijmegen The Netherlands; ^2^ Centre of Observations, Impacts, Energie, MINES Paris Tech, PSL University Sophia Antipolis France; ^3^ Environment Department, University of York, Heslington York United Kingdom; ^4^ Amsterdam Institute for Global Health and Development Amsterdam The Netherlands

**Keywords:** Exposure**–**response relationship, Ecotoxicity, Species sensitivity distribution, Biodiversity metric, Intraspecies variation

## Abstract

Chemical pollution of surface waters is considered an important driver for recent declines in biodiversity. Species sensitivity distributions (SSDs) are commonly used to evaluate the ecological risks of chemical exposure, accounting for variation in interspecies sensitivity. However, SSDs do not reflect the effects of chemical exposure on species abundance, considered an important endpoint in biological conservation. Although complex population modeling approaches lack practical applicability when it comes to the routine practice of lower tier chemical risk assessment, in the present study we show how information from widely available laboratory toxicity tests can be used to derive the change in mean species abundance (MSA) as a function of chemical exposure. These exposure–response MSA relationships combine insights into intraspecies exposure–response relationships and population growth theory. We showcase the practical applicability of our method for cadmium, copper, and zinc, and include a quantification of the associated statistical uncertainty. For all 3 metals, we found that concentrations hazardous for 5% of the species (HC_5_s) based on MSA relationships are systematically higher than SSD‐based HC_5_ values. Our proposed framework can be useful to derive abundance‐based ecological protective criteria for chemical exposure, and creates the opportunity to assess abundance impacts of chemical exposure in the context of various other anthropogenic stressors. *Environ Toxicol Chem* 2020;39:2304–2313. © 2020 The Authors. *Environmental Toxicology and Chemistry* published by Wiley Periodicals LLC on behalf of SETAC.

## Graphical abstract

Workflow for computing the Mean Species Abundance (MSA) relationship using exposure‐response data. Species‐specific exposure‐response curves for survival and reproduction endpoints are combined into a single exposure‐abundance curve. Multiple species‐specific exposure‐abundance curves are then aggregated into one MSA relationship.

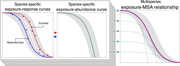

## INTRODUCTION

Chemical pollution of surface waters is considered an important driver of the deterioration of freshwater ecosystems (Ginebreda et al. 2014; Malaj et al. 2014; Bernhardt et al. 2017). In this context, species sensitivity distributions (SSDs) are commonly applied to assess the aquatic risks of chemicals (Posthuma et al. 2001; de Zwart and Posthuma 2005). The SSDs inform on the relative sensitivity of species, with each one represented by a single point on their specific exposure**–**response curve, for example, the no‐observed‐effect concentration (NOEC) or the 10% or 50% effective concentration (EC10 or EC50). Generally, the SSD describes the change in the potentially affected fraction of species over an exposure gradient (Posthuma et al. 2001). Derived from standard laboratory toxicity data, SSDs are suitable for high‐throughput lower tier assessments of aquatic risks, and are commonly used in regulatory settings to derive surface water quality standards or assess ecological risks (Posthuma et al. 2001).

The exact ecological interpretation of the fraction of species affected is, however, not straightforward, because population impacts, such as changes in species abundance, are not explicitly addressed. Arguably, the most realistic approaches to assess the impacts of chemical exposure on a community level are mesocosm experiments in which ecosystem structure or function are monitored over time (Iwasaki et al. 2018) with diagnostic assessments of chemical pollution in surface waters as complex but complete approaches. Such monitoring processes are, however, costly and time consuming. Modeling approaches have been proposed instead to integrate biological interactions and indirect ecological effects in chemical risk assessment practice (Naito et al. 2003; Galic et al. 2010; Forbes et al. 2011; de Laender et al. 2013; Gredelj et al. 2018). The gain in ecological relevance of these more complex modeling approaches comes with an inevitable loss of practical applicability, due to large data requirements for parameterization (Hendriks 2013). For example, the model proposed by Gredelj et al. (2018) requires extensive local‐ and chemical‐specific data for parameterization. As a consequence, risk assessments performed under European Union regulation 793/93/EC (European Commission 1993) are rarely based on food web models (Galic et al. 2010; de Laender et al. 2013).

Considering that more complex ecological models cannot be easily used in the routine practice of lower‐tier chemical risk assessment, there is a strong need for alternative approaches to estimate ecosystem‐level responses to chemical pollution (e.g., de Vries et al. 2010; Beaudouin and Péry 2013; de Laender et al. 2014). A metric that is commonly applied in ecological assessments is the mean species abundance (MSA; Janse et al. 2015; Newbold et al. 2016; Schipper et al. 2016). The MSA expresses the mean abundance of species in disturbed conditions relative to their abundance in undisturbed habitat (Alkemade et al. 2009; Benítez‐López et al. 2010; Janse et al. 2015; Schipper et al. 2019). Thus the MSA incorporates differences in both inter‐ and intraspecies abundance responses to an environmental stressor. The MSA responses are derived for a wide variety of environmental stressors, including land conversion, road disturbance, hunting, climate change, eutrophication, and dam construction, but up to now, they have not been used for chemical pollution (Alkemade et al. 2009; Benítez‐López et al. 2010; Janse et al. 2015).

We present a new method to derive relationships between chemical exposure and MSA, combining exposure–response model theory and traditional population growth concepts. Our method specifically aims to balance the low data requirements of SSDs that promote their high‐throughput applicability, while still improving the ecological interpretability of the assessment. In fact, our method makes use of the full exposure–response curve derived from laboratory tests, in contrast to SSDs, which utilize only one point of that curve. As a result, a priori management decisions on the relevant endpoints to base the SSD on, for example, the EC50 or EC10, do not have to be made. Our method follows 3 steps. First, species‐specific exposure–response relationships for reproduction and survival were fitted onto toxicity data extracted from the literature. Second, species‐specific responses for survival and reproduction were converted to responses for abundance. Third, the species‐specific exposure–abundance relationships were combined into an exposure–response MSA relationship (MSAR). A case study was then carried out to evaluate the procedure with 3 metals (cadmium [Cd], copper [Cu], and zinc [Zn]), using probabilistic simulations to also explore the influence of uncertainty associated with the exposure–response relationships. Lastly, we show the potential application of MSARs in lower tier assessments of aquatic risks by comparing the concentrations hazardous for 5% of the species (HC_5_s) of Cd, Cu, and Zn extracted from SSDs and MSARs, based on the same underlying data.

## MATERIALS AND METHODS

### MSAR framework

Exposure–response relationships for survival and reproduction were established using a 2‐parameter log‐logistic model, which is commonly used to fit sigmoidal exposure–response curves (Weiss 1997; Gadagkar and Call 2015). An example for reproduction exposure–response relationships is shown by Equation 1.

(1) $${\hat{Y}}_{\text{rep}}=1-\frac{1}{\left[1+{\left(\frac{C}{\text{EC}50}\right)}^{\beta }\right]}$$

where ${\hat{Y}}_{\text{rep}}$ represents the expected response fractions at exposure concentration c, EC50 depicts the half‐maximal effective concentration for reproduction, and $\beta_{\text{rep}}$ represents the slopes at the steepest part of the curve, also called the Hill slope (Gadagkar and Call 2015).

Depending on the exposure concentration, either or both survival and reproduction probabilities are lowered, altering the fecundity of the species studied and ultimately affecting its population abundance. Hendriks et al. (2005) derived a logistic exposure–response function describing the change in lifetime fecundity, that is, the average number of offspring/surviving adult, in exposed populations ($R_{0}(c))$ compared with their nonexposed equivalents ($R_{0}(0)$), as shown in Equation 2.

(2) $$\frac{R_{0}\left(c\right)}{R_{0}\left(0\right)}=\left(1-{\hat{Y}}_{\text{rep}}\right)\times (1-{\hat{Y}}_{\text{surv}})\;=\frac{1}{\left[1+{\left(\frac{C}{EC50}\right)}^{\beta_{\text{rep}}}\right]}\;\times \;\frac{1}{\left[1+{\left(\frac{C}{LC50}\right)}^{\beta_{\text{sur}v}}\right]}$$

where ${\hat{Y}}_{\text{rep}}$ and ${\hat{Y}}_{\text{surv}}$ are the expected response fractions at exposure concentration c for survival and reproduction. Under the assumption of logistic growth, the intrinsic rate of increase (*r*) is a function of the generation time *T*_g_ and lifetime fecundity $R_{0}$ (Equation 3; Pielou 1969; May 2001).

(3) $$r=\frac{\ln(R_{0})}{T_{g}}$$

Equation 3 holds under the assumption that each age class is affected to the same extent. By combining Equations 2 and 3, the intrinsic rate of increase of exposed r(c) versus nonexposed r(0) populations can be determined from Equation 4 (Hendriks and Enserink 1996). This equation specifically holds under the assumption that *T*_g_ is not affected by contaminants, and that a constant bioavailability exists across field and various test conditions (Hendriks et al. 2005).

(4) $$\frac{r\left(c\right)}{r\left(0\right)}=\frac{\ln\left(R_{0}\left(c\right),)\right){T_{g}}^{-1}}{\ln\left(R_{0}\left(0\right),)\right){T_{g}}^{-1}}=\frac{-\ln\left(1+{\left(\frac{C}{EC50}\right)}^{\beta_{\text{rep}}}\right)-\ln\left(1+{\left(\frac{C}{LC50}\right)}^{\beta_{\text{surv}}}\right)}{\ln(R_{0}(0))}+1$$

As shown both empirically (Hendriks et al. 2005) and analytically (Hakoyama et al. 2000), the decrease in a population's intrinsic rate of increase equals a proportional decrease in its carrying capacity (*K*). Thus r(c)/r(0) and K(c)/K(0) ratios can be considered equivalent. If exposure–abundance ratios (K(c)/K(0)) or exposure–population growth ratios (r(c)/r(0)) were directly available (e.g., from toxicity tests on algae), these were used in the MSAR method by fitting abundance or population growth data via Equation 5:

(5) $$\frac{K\left(c\right)}{K\left(0\right)}=\frac{r\left(c\right)}{r\left(0\right)}=\frac{-\ln\left(1+{\left(\frac{C}{C_{50}}\right)}^{\beta }\right)}{\ln(R_{0}(0))}+1$$

Because the carrying capacity determines the theoretical maximum population size, that is, the species‐specific abundance (van Gils et al. 2004), species‐specific K(c)/K(0) ratios can be aggregated into one overarching MSAR via Equation 6 (Alkemade et al. 2009; Benítez‐López et al. 2010; Janse et al. 2015).

(6) $$\mathrm{MSA}\left(c\right)=\frac{1}{n}\sum_{i=1}^{n}\frac{{K\left(c\right)}_{i}}{{K\left(0\right)}_{i}}$$

where MSA(c) is the mean abundance of all species at concentration c, expressed as a ratio from 0 to 1, and n is the number of species included in its derivation. The resulting MSA curve represents the relationship between the chemical exposure and the MSA, and is referred to as the MSAR.

**Figure 1:**
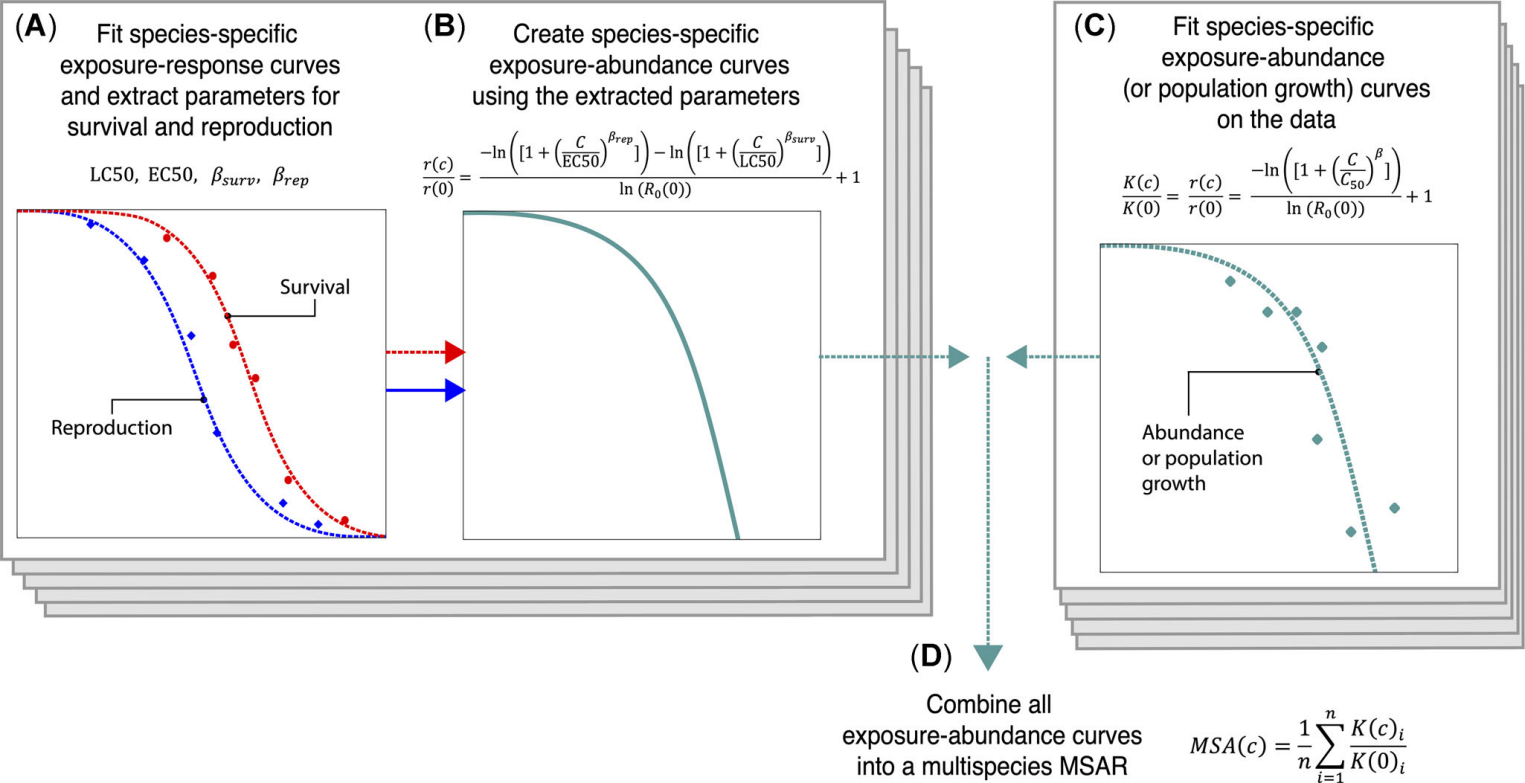
Workflow to compute deterministic mean species abundance relationships (MSARs). The entire process can be separated into 4 consecutive steps. (**A**) A 2‐parameter log‐logistic model (Equation 1) is fitted to exposure–response data for survival and reproduction, thereby obtaining the corresponding exposure–response parameters (median lethal or effect concentration (LC50 or EC50) and $\beta_{\text{surv}}$ or $\beta_{\text{rep}}$). (**B**) These parameters are combined with the undisturbed lifetime fecundity (*R*_0_; Equation 4), to create species‐specific exposure–abundance curves. (**C**) Exposure–response data on abundance or population growth are fitted to Equation 5, resulting in their respective continuous exposure–abundance curves. (**D**) All exposure–abundance relationships are combined into one MSAR (Equation 6).

The L(E)C50 and *β* values 
were derived using the NLS function available in R (R Core Development Team 2015). An overview of the workflow for deriving MSARs is shown in Figure 1.

### Case study

We illustrate the applicability of our approach for 3 metals, Cd, Cu, and Zn. To do so, we gathered studies reporting species‐specific chronic exposure–response data on reproduction and survival (animal species), or on abundance and population growth (algae). From survival and reproduction studies, we recorded the mean response/concentration tested, and used Equation 2 to extract all required exposure–response model parameters (LC50 or EC50 and *β*_surv_ or *β*_prep_). Selecting studies for the exposure–response data on survival was relatively straightforward, because most studies directly describe the number of surviving individuals in each exposure–response group. Reproduction‐related endpoints, however, can be measured at multiple stages in the reproductive cycle. To match species‐specific exposure–response survival and reproduction data, and to have sufficient data on multiple trophic levels, we assumed that data on a selective part of the reproduction cycle (e.g., hatchability of fertilized eggs, number of gravid adult females) would have approximate effects on the full reproduction cycle. All studies and data points used are included in the Supplemental Data (Section 1, Figures S1–S5), as is the endpoint categorization for survival and reproduction (Supplemental Data, Section 1, Tables S1–S3).

In the fitting of the exposure–response curves, we ignored potential differences in bioavailability in laboratory and field studies, associated with varying physicochemical circumstances, for example, pH and temperature. Such bioavailability differences can be addressed via biotic ligand models (Garman et al. 2020), but data limitations in both laboratory and field studies currently hamper us from doing so comprehensively.

Experimental data on the undisturbed lifetime fecundity ($R_{0})$ of individual species were also collected. For clonal species such as algae, a lifetime fecundity of 2 was assumed (Hendriks 2007). When no experimental $R_{0}$ values were available, we applied allometric relationships between body weight and intrinsic rate of increase *r*, and between body weight and generation time *T*_g_ (Blueweiss et al. 1978; Hendriks 2007). The $R_{0}$ was then estimated using Equation 3 (see Supplemental Data, Section 2, Equations S1 and S2).

### MSAR for risk assessment

To evaluate the potential of the MSAR approach for lower tier assessments of aquatic risks, we derived the hazardous concentration for 5% of the species (HC_5_) for each metal individually, using both MSAR and SSD curves. A lower percentile of a compound's SSD built from NOEC data for example, such as the HC_5_, or more specifically the HC_5_–NOEC, is often used to help in deriving a protective environmental quality standard for regulatory uses (Posthuma et al. 2001). Our SSDs were fitted on EC10 values extracted from the same exposure–response data underlying the MSAR curve, allowing a direct comparison between both methods. In addition to HC_5_–EC10 values derived from these (EC10‐based) SSDs, we also compared our MSAR‐based HC_5_ values with HC_5_–NOEC values from regulatory reports (European Chemicals Agency 2007; European Chemicals Bureau 2007, 2008). The comparison with regulatory values can provide insights into the overall sensitivity of the collected data, the SSDs derived from our data set, and the MSAR framework. Finally, we calculated the MSA loss for each chemical individually at the concentration equal to the HC_5_–EC10 extracted from the SSDs compiled in the present study. The R package ssdtools was used for fitting log‐logistic SSD curves and quantifying the uncertainty (Thorley et al. 2018). All calculations, simulations, and statistical analyses were performed in R Ver 3.4.1 (R Core Development Team 2015).

### Uncertainty quantification

We quantified the statistical uncertainty in the MSAR by accounting for residual error in the exposure–response model fit. In all cases, exposure–response curves were sampled by utilizing the function predictNLS included in the R package propagate, which relies on second‐order Taylor expansion in a Monte Carlo approach to simulate the uncertainty around an optimal fit (Tellinghuisen 2001; Spiess 2018). The function returns the optimal model fit and associated confidence interval (CI). We modified the function to return iteration outcomes of the uncertainty simulation one at a time, each representing one possible exposure–response curve to be used in the next steps. In the case of survival and reproduction data, the exposure–response curves were combined into an exposure–abundance relationship (Equation 4). When abundance or population growth data were available, the uncertainty in exposure–abundance fit was quantified directly, by fitting Equation 5 using the NLS function and sampling a possible exposure–response curve using the modified predictNLS function. After exposure–abundance curves were obtained for all *n* species, the *n* exposure–abundance curves were aggregated into a single possible MSAR. This entire process was repeated 1000 times in a Monte Carlo approach, thereby creating 1000 possible MSAR curves. We used the 95% CIs to visualize the simulated uncertainty around the deterministic MSAR curve.

## RESULTS

### *exposure*–*abundance relationships*

**Figure 2:**
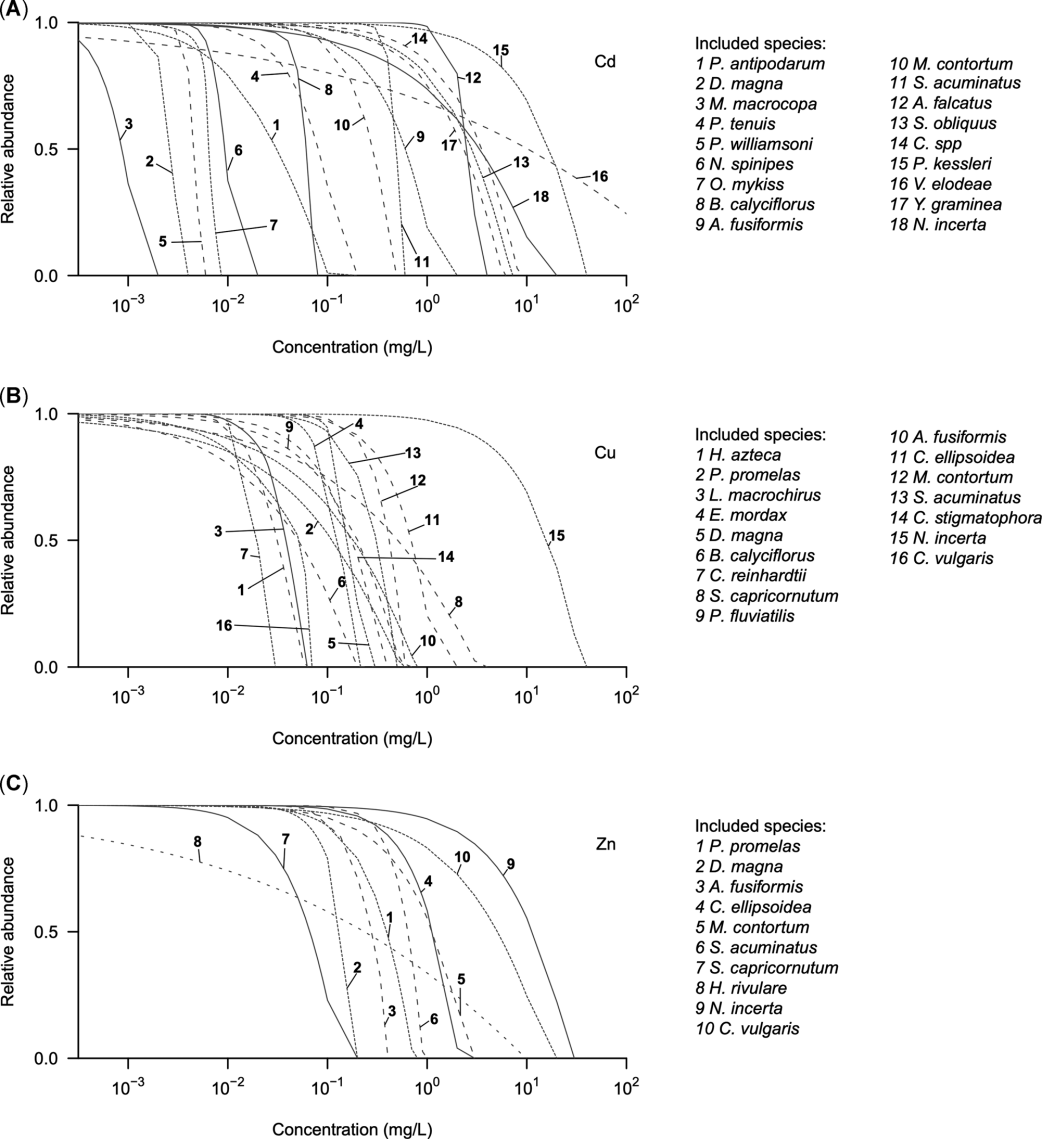
Species‐specific exposure–abundance curves for (**A**) cadmium (Cd), (**B)** copper (Cu), and (**C**) zinc (Zn).

We obtained exposure–response information for Cd (*n*_*species*_ *=* 18), Cu (*n*_*species*_ *=* 16), and Zn (*n*_*species*_ *=* 10) and computed exposure–abundance curves based on survival and reproduction or abundance endpoints (Figure 2). For Cd, the concentration resulting in a 50% abundance loss was on average approximately 10 times higher when only survival endpoints were considered instead of both survival and reproduction endpoints. Although this difference was smaller for Cu and Zn, still 70% of the species had survival‐based exposure–abundance curves outside the 95% CI around the curves based on both survival and reproduction.

### Mean species abundance relationships

**Figure 3:**
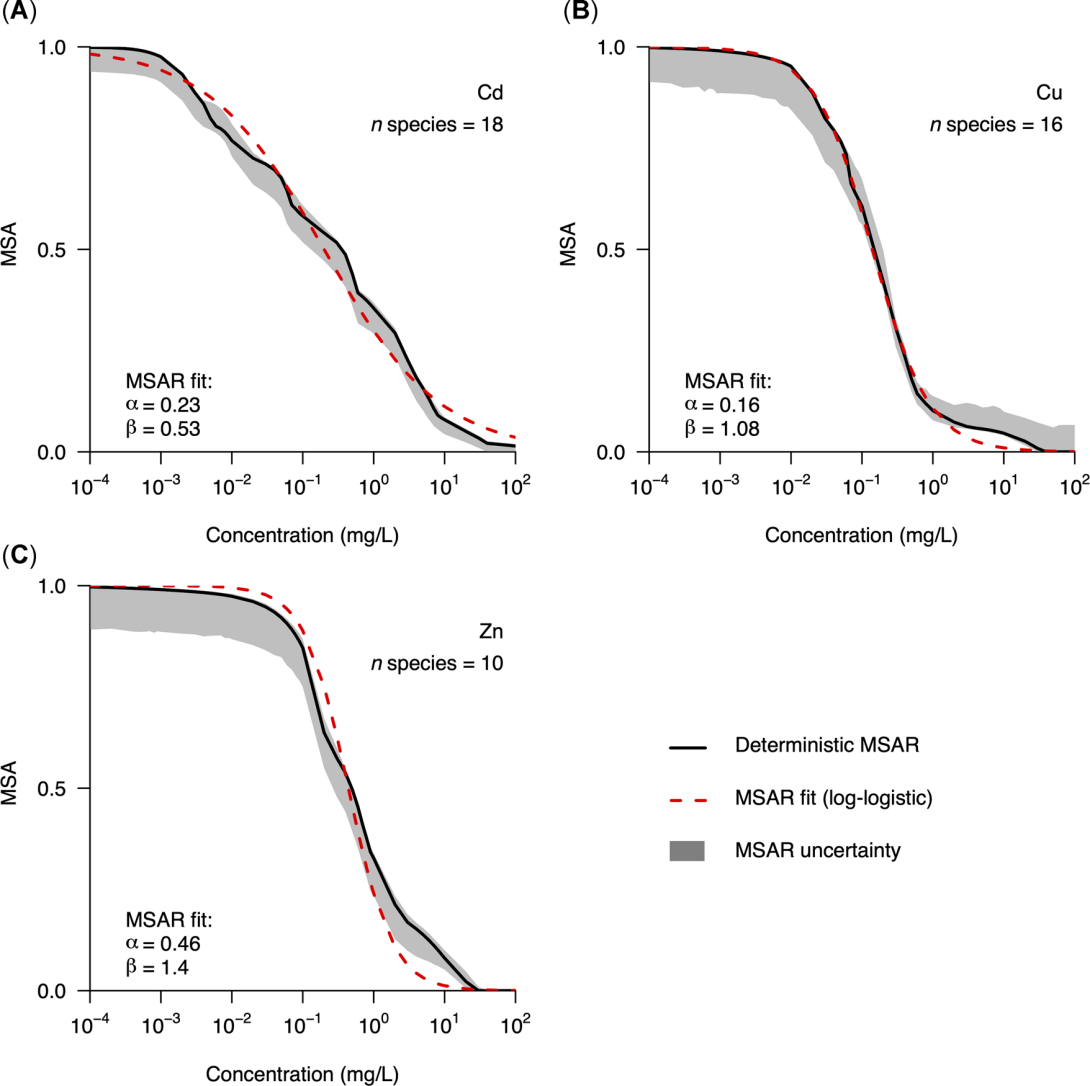
Mean species abundance relationship (MSAR) for (**A**) cadmium (Cd), (**B**) copper (Cu), and (**C**) zinc (Zn). Black solid lines represent the deterministic MSAR, computed directly from the exposure–abundance relationships shown in Figure 2. The gray areas surrounding the MSAR show 95% confidence intervals of the simulated data (1000 iterations). Red dashed lines show the log‐logistic models fitted through the deterministic MSAR.

The species‐ and chemical‐specific exposure–abundance relationships (Figure 2) were subsequently combined into MSAR curves for Cd (Figure 3A), Cu (Figure 3B), and Zn (Figure 3C) by making use of Equation 6 (see Figure 1D). Besides the deterministic MSAR curves (solid black lines, Figure 3), the 95% CIs of the simulated data are presented (gray areas, Figure 3).

**Figure 4:**
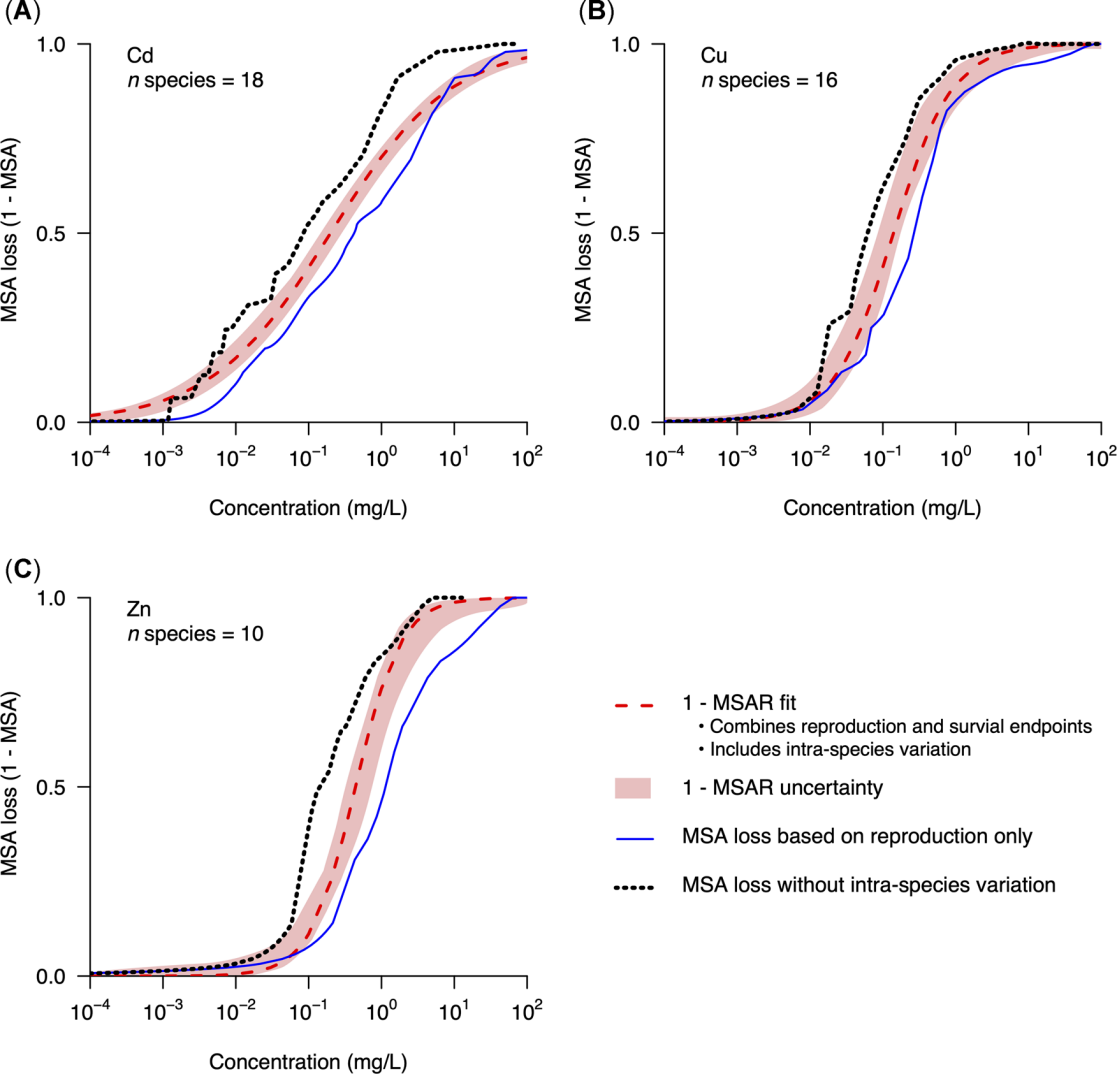
Mean species abundance (MSA) loss for (**A**) cadmium (Cd), (**B**) copper (Cu), and (**C**) zinc (Zn). The red dashed lines indicate the losses in MSA determined by 1 – MSA Relationship (MSAR; log‐logistic fit). The red areas surrounding the MSAR show the 95% confidence intervals of the simulated data (1000 iterations). The blue solid lines represent the MSA loss determined from solely reproduction (i.e., excluding survival). The black dotted lines represent the MSA loss for both reproduction and survival but excluding all intraspecies variation (i.e., ignoring the exposure–response slopes).

Next, we tested to what extent different assumptions for the definition of MSARs can influence the derived curve (Figure 4). For this purpose, we derived 2 alternative MSAR curves. The first excludes the effect of survival on the total MSAR curve by solely taking the reproduction data into consideration. The difference between the default MSAR curve including the effects on both reproduction and survival and the alternative MSAR curve solely based on reproduction varied among the 3 metals studied (Figure 4). This relative difference was quantified as the ratio between the parameters from the reproduction‐based exposure‐MSAR ($a_{\text{repro}}$ and $\beta_{\text{repro}}$) and those from the default exposure‐MSAR ($a_{\text{def}}$ and $\beta_{\text{def}}$; Figure 4). For Cd, these ratios were $a_{\text{repro}}/a_{\text{def}}$ = 1.17 and $\beta_{\text{repro}}/\beta_{\text{def}}$  = 0.84. For Cu, the relative differences were larger ($a_{\text{repro}}/a_{\text{def}}$ = 2.70 and $\beta_{\text{repro}}/\beta_{\text{def}}$ = 0.78). The largest differences were observed for Zn ($a_{\text{repro}}/a_{\text{def}}$ = 7.95 and $\beta_{\text{repro}}/\beta_{\text{def}}$ = 0.64).

We also compared the MSAR with a second alternative MSAR, determined under the assumption of no intraspecies variation (black solid lines, Figure 4). The MSAR that excludes all intraspecies variation was computed by considering infinitely large exposure–response slopes (*β* values), that is, using all‐or‐nothing exposure responses for the species included. Therefore, the resulting MSAR only depends on the EC50 and LC50 values for the individual species. The slopes for all exposure‐MSA curves that exclude intraspecies variation were 1.70 times (Cd), 1.69 times (Cu), and 1.24 times (Zn) larger than those of the default exposure‐MSA curve. In addition, the half‐maximal effective concentrations (a) for these curves (without intraspecies variation) were on average 2 times smaller for all 3 metals.

### MSAR for risk assessment

The MSAR‐based HC_5_ values are systematically higher (by a factor of 3.5–8.9) for the 3 metals compared with HC_5_–EC_10_ values derived from the SSDs we compiled, using the same data (Table 1). Comparing the regulatory HC_5_–NOEC values with the ones derived with EC10‐based SSD, differences by factors of 1 to 5.6 were obtained. We also calculated the percentage of MSA loss at the HC_5_–EC10. The MSA loss corresponding to the HC_5_–EC10 was 2.6% (95% CI: 0.1–2.9%) for Cd, 0.7% (95% CI: 0.0–1.0%) for Cu, and 1.8% (95% CI: 1.2–6.9%) for Zn. A visual presentation of the MSAR curves and EC10‐based SSDs for Cd, Cu, and Zn can be found in the Supplemental Data, Figure S6.

**Table 1:** Concentration hazardous for 5% of the species (HC_5_) values extracted from the exposure–mean species abundance (MSA) relationships and the 10% effect concentration (EC10)‐based species sensitivity distribution (SSD) curves for cadmium (Cd), copper (Cu), and zinc (Zn) based on the compiled data set presented in the Supplemental Data, Tables S1 to S3^a^

Metal	MSAR HC_5_ (μg/L)	SSD HC_5_‐EC10 (μg/L)	Regulatory SSD NOEC‐HC_5_ (μg/L)	Regulatory reference
Cd	0.89 (0.34–2.61)	0.10 (0.05–2.11)	0.38	European Chemicals Bureau 2007
Cu	9.42 (2.24–19.3)	1.31 (0.31–7.71)	7.30	European Chemicals Agency 2007
Zn	55.1 (11.1–69.9)	15.8 (6.37–51.2)	15.6	European Chemicals Bureau 2008

^a^ The 95% confidence intervals around the HC_5_ are in parentheses. For comparison, regulatory NOEC‐HC_5_ values are also presented. All values were extracted from log‐logistic fits though the corresponding data. MSAR = MSA relationship; NOEC = no‐observed‐effect concentration.

## DISCUSSION

We have shown how the MSAR can be derived by combining single‐species concentration–response data with established population growth concepts. Multiple species‐specific exposure–abundance curves were summarized into a single MSAR, providing insights into the overall decline of species abundance on a community level (Alkemade et al. 2009; Benítez‐López et al. 2010; Janse et al. 2015).

### Interpretation of results

The case study with 3 metals provides first insights into the underlying factors driving the MSAR. By disregarding the intraspecies variation (i.e., using a single point from the full exposure–response curves) and by mixing data irrespective of endpoint, the SSD has a major advantage in being far less constrained by data availability than the MSAR. However, these aspects also make SSDs harder to interpret and possibly lower their ecological relevance. Indeed, exclusion of all intraspecies variation (Figure 4), results in steeper and more sensitive MSARs, compared with the default MSARs computed. The MSARs only based on the most sensitive endpoint (i.e., reproduction), however, were shown to be far less steep and less sensitive in general. The results we present, combined with the given nature of the equations used to compute the MSAR (Equations 4 and 5), provide a first indication that: 1) depending on the chemical studied, the inclusion of intraspecies variation (i.e., species‐specific exposure‐response βs) might be highly relevant in accurately determining the MSAR; and 2) depending on balance in the sensitivity to either reproduction or survival, the inclusion of 2 endpoints can be important in accurately determining the MSAR. Finally, an MSAR based on a single endpoint and without the inclusion of intraspecies variation will mostly follow the cumulative distribution of the EC50 values (see explanation provided in the Supplemental Data, Section 4). Therefore, SSDs based on chronic EC50 values might be considered a good proxy of the MSA loss for chemicals with little intraspecies variation in sensitivity and with a single most sensitive endpoint.

In the derivation of HC_5_ values, we found that our MSAR‐based method provides higher concentration values compared with the SSD‐based methods. This is true both for the regulatory SSD‐based HC_5_–NOEC values and for the HC_5_–EC10 values extracted from our own SSDs. An SSD is built from single species‐specific metrics that represent either the concentration not affecting the species at all (NOEC) or the concentration at which 10% is affected (EC10). The MSAR approach, however, uses the entire exposure–response curve, ultimately resulting in a less conservative overall MSAR curve. Note, however, that the SSDs presented in our study are based on a limited sample of the available ecotoxicity data for the 3 metals included and do not represent the common data requirements when SSDs are used in a formal regulatory context (Posthuma et al. 2001). Although regulatory HC_5_–NOEC values as applied in the European regulatory context fall within the 95% CIs of the HC_5_–EC_10_ values derived in the present study, the numbers of species we were able to include because of the MSAR requirements were 18 (Cd), 16 (Cu), and 10 (Zn), compared with 44 (Cd), 28 (Cu), and 18 (Zn) used in the regulatory reports (European Chemicals Agency 2007; European Chemicals Bureau 2007, 2008).

### Limitations

Although the case study with 3 metals helped us demonstrate the potential utility of the MSAR for decision support purposes, additional research is required to further explore and extend the practical applicability of our findings. First, our MSARs for the 3 metals did not consider potential differences in bioavailability between different laboratory exposures as well as between laboratory and field conditions. For practical assessments, it is imperative to account for bioavailability differences between laboratory and field, as discussed in Garman et al. (2020). Second, for some species we utilized reproduction endpoints that only partially quantify the effect on the reproduction cycle, due to a lack of more extensive reproduction data (see the Supplemental Data, Tables S1–S3). Third, we quantified the uncertainty due to suboptimal model fit, but we did not quantify the statistical uncertainty associated with the values used for lifetime fecundity $R_{0}$ due to a lack of empirical data. As an alternative to the use of experimental $R_{0}$ values, species‐specific allometric relationships can be applied, as we did for the majority of species in our case study, adding further uncertainty to the MSAR response. Note, however, that the intrinsic rates of increase (*r*) and generation time (*T*_g_) follow opposite scaling exponents (in relation to body mass; see Supplemental Data, Section 3, Table S4), so that the variation in $R_{0}$ is expected to be largely independent of body mass (Hendriks 2007). Fourth, we assumed that the generation time (*T*_g_) was not affected by contaminants (Equation 4; Hendriks et al. 2005). Theoretically, a simple modification to Equation 4 would allow for the inclusion of the effect of the chemical exposure on *T*_g_. However, data and theory quantifying this effect are sparse for specific chemicals. Fifth, we assumed that r(C)/r(0) is proportional to K(C)/K(0). Although many studies report relative changes in intrinsic growth rate (r(C)/r(0)) to be proportional to those in carrying capacity K(C)/K(0); Hakoyama et al. 2000; Nakamaru et al. 2003; Hendriks et al. 2005; Hilbers et al. 2018), there are also studies arguing against such a relationship (e.g., Bell 1990; Underwood 2006). Sixth, the calculations also disregard ecologically relevant processes like interspecies interactions (e.g., competition), which may lead to an underestimation of the impact of chemical stressors on a community level (de Laender et al. 2008). Finally, a comparison of MSAR results with insights and data obtained in the field or from mesocosm studies (e.g., Iwasaki et al. 2018), and from alternative population models (e.g., Gredelj et al. 2018) could help to extend the confidence in the method proposed. In conclusion, although our MSAR method could be improved in various ways, most improvements show exciting opportunities to even further increase the ecological relevance of the method.

### Practical implementation

For a practical implementation of the MSAR in ecological risk assessment, data on the full (chronic) exposure–response curves are required for several endpoints, that is, survival, reproduction or abundance, of a sufficiently large set of species. In addition, the experimental conditions (e.g., pH, dissolved organic carbon, alkalinity) need to be defined to consider the bioavailability of metals. These requirements may, however, potentially hamper a successful implementation of our proposed method for a larger set of chemicals. Minimizing the MSAR data requirements can be realized in 2 ways. First, reproduction was clearly the most sensitive endpoint in our calculations. As a consequence, exclusion of survival data will most likely only slightly change MSARs for most chemicals. Second, instead of fitting a relationship on the species‐specific exposure–response data (for reproduction), the parameters describing this relationship, that is, EC50 and slope *β*, may be derived from other sources. For instance, reproduction EC50 values for many chemicals can be obtained from databases such as ECOTOX (US Environmental Protection Agency 2019), or derived with quantitative structure–activity relationships. Default value(s) for β may be derived from meta‐analyses, such as the one conducted by Smit et al. (2001).

In conclusion, by using information on population growth and species‐specific exposure–response curves derived from laboratory data, our method can be applied to derive chemical impacts and concentration limits on a population level. In doing so, the MSAR could be used to advance the tier 2 risk assessment to an ecologically relevant indicator (European Food Safety Authority 2013), that is, the MSAR. Our MSAR framework might offer a middle ground between complex population modeling approaches, which lack practical applicability on a large scale, and chemical risk assessment methods that might provide inadequate insights into ecological consequences (Calow and Forbes 2003; Galic et al. 2010; Forbes et al. 2011).

## Supplemental Data

The Supplemental Data are available on the Wiley Online Library at https://doi.org/10.1002/etc.4850.

## Author Contributions Statement

R. Oldenkamp conceived the concept. S. Hoeks, R. Oldenkamp, and M.A.J. Huijbregts designed the approach. S. Hoeks performed the analysis, created the figures, and wrote the manuscript. M. Douziech and S. Hoeks collected the data. M. Douziech, A.J. Hendriks, R. Oldenkamp, and M.A.J. Huijbregts provided technical and editorial assistance.

## Supporting information

This article includes online‐only Supplemental Data.

Supporting information.

## Data Availability

Data, associated metadata, and calculation tools are available from the corresponding author (s.hoeks@science.ru.nl).
