# Supplementary material for: Mean Species Abundance as a Measure of Ecotoxicological Risk
Source: Environ Toxicol Chem. 2020 Sep 16;39(11):2304–13. doi: 10.1002/etc.4850 (PMC7693057; doi:10.1002/etc.4850)
Supplement: Supplementary file 1 — Supporting information. [file ETC-39-2304-s001.docx]

**Supporting Information**

**Mean Species Abundance as a Measure of Ecotoxicological Risk**

Number of pages: 16

Number of figures: 6

Number of tables: 4

Number of equations: 4

**Table of contents:**

- Section 1. Case study data **page 2-9**
  - Table S1: Exposure-response data details for the cadmium (Cd) page 2
  - Table S2: Exposure-response data details for the copper (Cu) page 3
  - Table S3: Exposure-response data details for the zinc (Zn) page 4
  - Figure S1: Exposure-response curves and data for cadmium (Cd) page 5
  - Figure S2: Exposure-response curves and data for cadmium (Cd) page 6
  - Figure S3: Exposure-response curves and data for copper (Cu) page 7
  - Figure S4: Exposure-response curves and data for copper (Cu) page 8
  - Figure S5: Exposure-response curves and data for zinc (Zn) page 9

### Section 2. Population growth parameters page 10

- - Equation S1 page 10
  - Equation S2 page 10

### Section 3. Supplementary information results page 11-12

- - Figure S6: MSAR and SSD curve comparison page 11
  - Table S4: Range in $R_{0}$ explained page 12
- Section 4: Simplifying the MSAR **page 13**
  - Equation S3 page 13
  - Equation S4 page 13
- SI References **page 14-16**

**Section 1. Case study data**

This section includes the references, data and experimental details used to derive the MSARs as well as the SSDs for cadmium (Cd), copper (Cu), and zinc (Zn). Table S1, S2 and S3 show the experimental details as well as the references used to extract the data from. Figure S1 to S5 show the raw exposure-response data points for survival, reproduction, abundance and population growth endpoints. In addition, Figure S1 to S5 show the exposure-response models and the exposure-abundance models fitted on the corresponding data points.

**Table S1:** Exposure-response data details for the cadmium (Cd) case study. Experimental details directly extracted from the references.

| **Reference** | **Species** | **Tax_class** | **Endpoint classification** | **Endpoint specified in paper** | **Exposure hours** | **pH** | **Temperature** |
| --- | --- | --- | --- | --- | --- | --- | --- |
| Snell et al. 1992 | Brachionus Calyciflorus | Monogononta | Abundance/Growth | Rate of increase | 48 | 7.5 | NA |
| Magdaleno et al. 2013 | Ankistrodesmus fusiformis | Chlorophyceae | Abundance/Growth | Pop. Growth rate | 96 | NA | 24 |
| Prasad & Prasa 1982 | Ankistrodemus falcatus | Chlorophyceae | Abundance/Growth | Optical density | 240 | NA | 23.1 |
| Prasad & Prasa 1982 | Scenedesmus obliquus | Chlorophyceae | Abundance/Growth | Optical density | 240 | NA | 23.1 |
| Prasad & Prasa 1982 | Chlorococcum spp | Chlorophyceae | Abundance/Growth | Optical density | 240 | NA | 23.1 |
| Ngo et al. 2009 | Parachlorella kessleri | Trebouxiophyceae | Abundance/Growth | Abundance | 120 | 7 | 26 |
| Azevedo and Cássio 2010 | Varicosporium elodeae | Leotiomycetes | Abundance/Growth | Abundance | 240 | 6 | 18 |
| Azevedo and Cássio 2010 | Ypsilina graminea | Ascomycota | Abundance/Growth | Abundance | 240 | 6 | 18 |
| Rachlin et al. 1983 | Navicula incerta | Bacillariophyceae | Abundance/Growth | Growth response | 99 | 8.0-8.8 | 19 |
| Magdaleno et al. 2013 | Monoraphidium contortum | Chlorophyceae | Abundance/Growth | Growth | 96 | NA | 24 |
| Magdaleno et al. 2013 | Scenedesmus acuminatus | Chlorophyceae | Abundance/Growth | Growth | 96 | NA | 24 |
| Sieratowicz et al. 2011 | Potamopyrgus antipodarum | Gastropoda | Reproduction | embryo number per female | 672 | 8.0-8.5 | 16 |
| Barata and Donald 2000 | Daphnia magna | Branchiopoda | Reproduction | Offspring Production | 504 | NA | 20 |
| Hatakeyama and Yasuno 1981 | Moina macrocopa | Branchiopoda | Reproduction | Mean brood size (offspring born/adult) | 480 | 7.5 | 23 |
| Indeherberg et al. 1999 | Polycelis tenuis | Rhabditophora | Reproduction | Decrease in reproductive allocation | 504 | 7.1-7.8 | 16 |
| Sieratowicz et al. 2011 | Prosopium williamsoni | Actinopteri | Reproduction | embryo number per female | 672 | 8.0-8.5 | 16 |
| Bengtsson and Bergström 1987 | Nitocra spinipes | Hexanauplia | Reproduction | Number of offspring produced | 312 | NA | 21 |
| Brown et al. 1994 | Oncorhynchus mykiss | Actinopterygii | Reproduction | Survival of eggs | 1176 | 7.5 | 15 |
| Sieratowicz et al. 2011 | Potamopyrgus antipodarum | Gastropoda | Survival | Mortality | 672 | 8.0-8.5 | 16 |
| Van Leeuwen et al. 1985 | Daphnia magna | Branchiopoda | Survival | Survival | 504 | 8.4 | 20 |
| Hatakeyama and Yasuno 1981 | Moina macrocopa | Branchiopoda | Survival | Mortality | 480 | 7.5 | 23 |
| Indeherberg et al. 1999 | Polycelis tenuis | Rhabditophora | Survival | Mortality | 504 | 7 | 16 |
| Brinkman and Vieira 2008 | Prosopium williamsoni | Actinopteri | Survival | Mortality | 720 | 6.8 | 9.5 |
| Bengtsson and Bergström 1987 | Nitocra spinipes | Hexanauplia | Survival | Survival | 504 | NA | 21 |
| Brown et al. 1994 | Oncorhynchus mykiss | Actinopterygii | Survival | Survival | 9000 | 7.5 | 15 |

**Table S2:** Exposure-response data details for the copper (Cu) case study. Experimental details directly extracted from the references.

| **Reference** | **Species** | **Tax_class** | **Endpoint classification** | **Endpoint specified in paper** | **Exposure hours** | **pH** | **Temperature** |
| --- | --- | --- | --- | --- | --- | --- | --- |
| Winner & Owen 1991 | Chlamydomonas reinhardtii | Chlorophyceae | Abundance/Growth | Cells/field | 72 | 8-8.3 | NA |
| Van der Heever & Grobbelaar 1996 | Selenastrum capricornutum | Chlorophyceae | Abundance/Growth | Changes in cell numbers | 72 | 7.5 | 23 |
| De Schamphelaere et al. 2006 | Brachionus Calyciflorus | Monogononta | Abundance/Growth | Rate of increase | 48 | 7.8 | 25 |
| Collvin 1984 | Perca Fluviatilis | Actinopteri | Abundance/Growth | Growth rate | 720 | 7.8 | 15.1 |
| Magdaleno et al. 2013 | Ankistrodesmus fusiformis | Chlorophyceae | Abundance/Growth | Pop. Growth | 96 | NA | 24 |
| Magdaleno et al. 2013 | Chlorella ellipsoidea | Trebouxiophyceae | Abundance/Growth | Pop. Growth | 96 | NA | 24 |
| Magdaleno et al. 2013 | Monoraphidium contortum | Chlorophyceae | Abundance/Growth | Pop. Growth | 96 | NA | 24 |
| Magdaleno et al. 2013 | Scenedesmus acuminatus | Chlorophyceae | Abundance/Growth | Pop. Growth | 96 | NA | 24 |
| Christensen et al. 1980 | Chlorella stigmatophora | Trebouxiophyceae | Abundance/Growth | Growth rate | 240 | NA | NA |
| Rachlin et al. 1983 | Navicula incerta | Bacillariophyceae | Abundance/Growth | Growth response | 99 | 8.0-8.8 | 19 |
| Rosko & Rachlin 1977 | Chlorella vulgaris | Trebouxiophyceae | Abundance/Growth | Growth response | 792 | NA | 21 |
| Othman & Pascoe 2002 | Hyalella azteca | Malacostraca | Reproduction | Number of gravid females | 840 | 7.9 | 22 |
| Pickering et al. 1977 | Pimephales promelas | Actinopteri | Reproduction | Mean No. Eggs/ female | 192 | NA | 20-26 |
| Rice & Harrison 1978 | Engraulis mordax | Actinopteri | Reproduction | Hatching | 32-37 | 8.17 | 16.8 |
| Dave 1984 | Daphnia magna | Branchiopoda | Reproduction | Progeny per female | 504 | 6.6-8.1 | 19.5-21 |
| Benoit 1974 | Lepomis macrochirus | Actinopterygii | Reproduction | Mean spawnings/female | 1000+ | 7.5 | 20 |
| Deaver Rodgers 1996 | Hyalella azteca | Malacostraca | Survival | Mortality | 240 | 7.9-8.2 | 20 |
| Van Leeuwen et al 1988 | Daphnia magna | Branchiopoda | Survival | Survival | 504 | 8.1 | 20 |
| Rice & Harrison 1978 | Engraulis mordax | Actinopteri | Survival | Mortality | 46 | NA | 16.8 |
| Mount 1968 | Pimephales promelas | Actinopteri | Survival | Survival | 7920 | 7.9 | 16-25.5 |
| Benoit 1974 | Lepomis macrochirus | Actinopterygii | Survival | Survival | 1000+ | 7.5 | 20 |

**Table S3:** Exposure-response data details for the zinc (Zn) case study. Experimental details directly extracted from the references.

| **Reference** | **Species** | **Tax_class** | **Endpoint classification** | **Endpoint specified in paper** | **Exposure hours** | **pH** | **Temperature** |
| --- | --- | --- | --- | --- | --- | --- | --- |
| Magdaleno et al. 2013 | Ankistrodesmus fusiformis | Chlorophyceae | Abundance/Growth | Pop. Growth | 96 | NA | 24 |
| Magdaleno et al. 2013 | Chlorella ellipsoidea | Trebouxiophyceae | Abundance/Growth | Pop. Growth | 96 | NA | 24 |
| Magdaleno et al. 2013 | Monoraphidium contortum | Chlorophyceae | Abundance/Growth | Pop. Growth | 96 | NA | 24 |
| Magdaleno et al. 2013 | Scenedesmus acuminatus | Chlorophyceae | Abundance/Growth | Pop. Growth | 96 | NA | 24 |
| Bartlett et al. 1974 | Selenastrum capricornutum | Chlorophyceae | Abundance/Growth | Pop growth from dry weight | 96 | 6.8-7.2 | NA |
| Hargreaves & Whitton 1976 | Hormidium rivulare | Trebouxiophyceae | Abundance/Growth | Growth rate as chlorophyll | NA | 7 | NA |
| Rachlin et al 1983 | Navicula incerta | Bacillariophyceae | Abundance/Growth | Growth response | 99 | 8.0-8.8 | 19 |
| Rosko Rachlin 1977 | Chlorella vulgaris | Trebouxiophyceae | Abundance/Growth | Growth response | 792 | NA | 21 |
| Benoit & Holcombe 1978 | Pimephales promelas | Actinopteri | Reproduction | Hatchability | 1344 | 7-8 | 25 |
| Perez & Hoang 2017 | Daphnia magna | Branchiopoda | Reproduction | Production of neonates | 504 | 7.6 | 22.6 |
| Perez & Hoang 2017 | Daphnia magna | Branchiopoda | Survival | Mortality | 504 | 7.6 | 22.6 |
| Bengtsson 1974 | Pimephales promelas | Actinopteri | Survival | Survival | 3600 | 7.5 | NA |

**
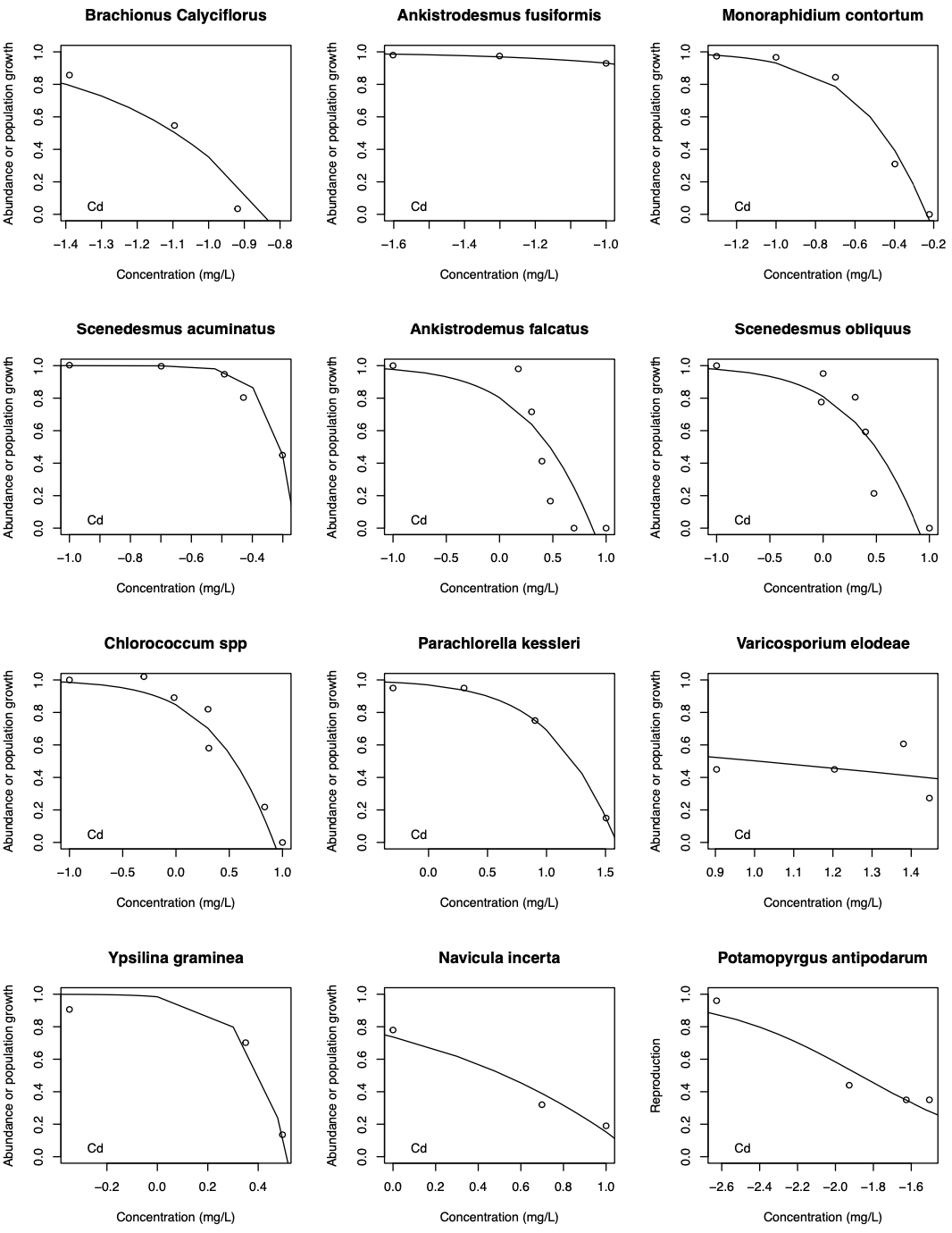
**

**Figure S1:** First part of the species-specific exposure-response data and curves obtained for Cadmium (Cd).

**
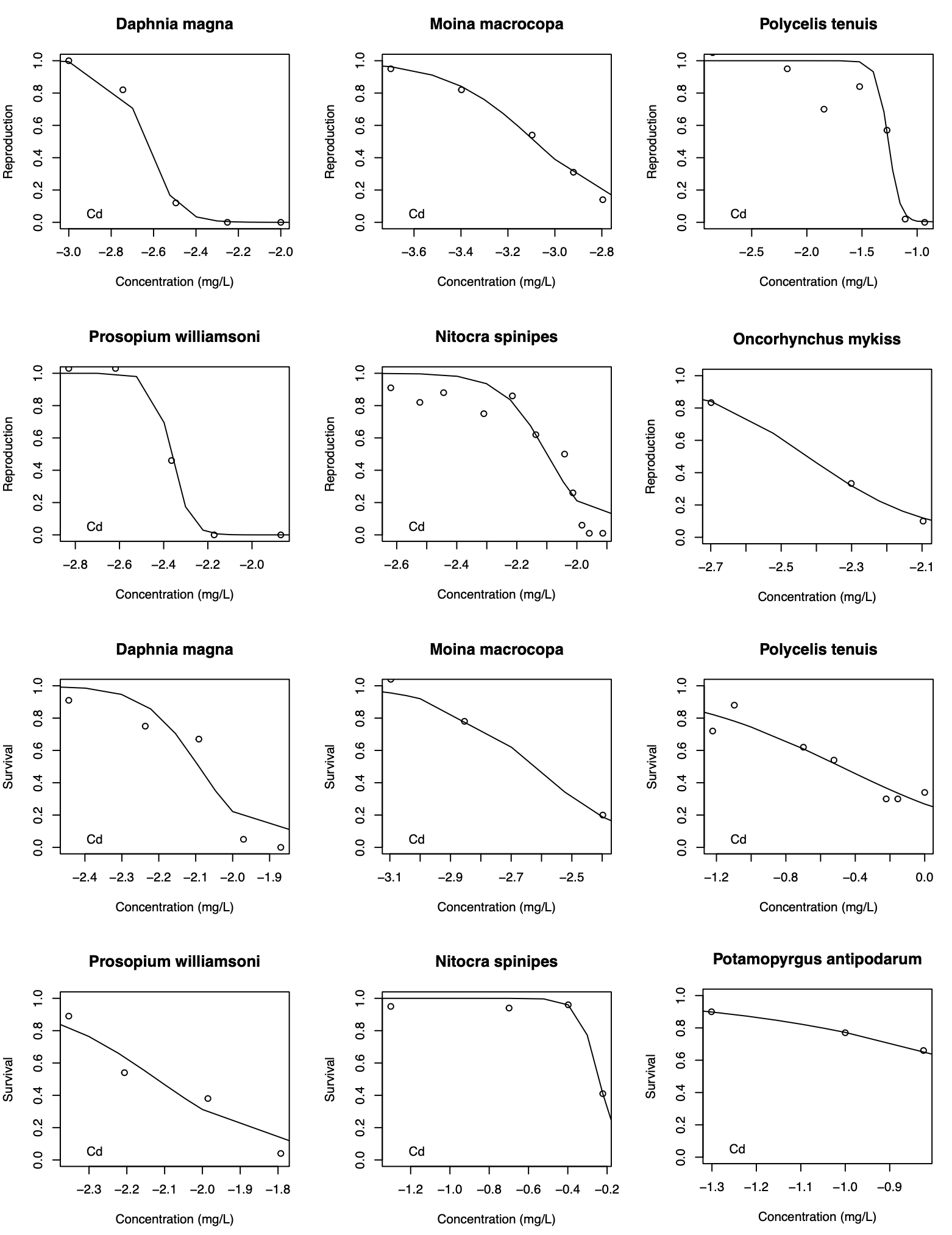
**

**
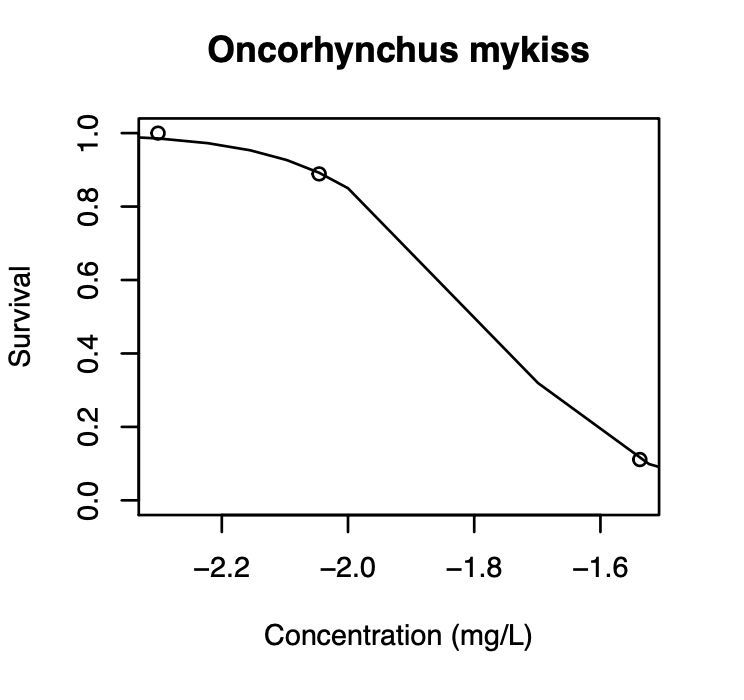
**

**Figure S2:** Second part of the species-specific exposure-response data and curves obtained for Cadmium (Cd).

**
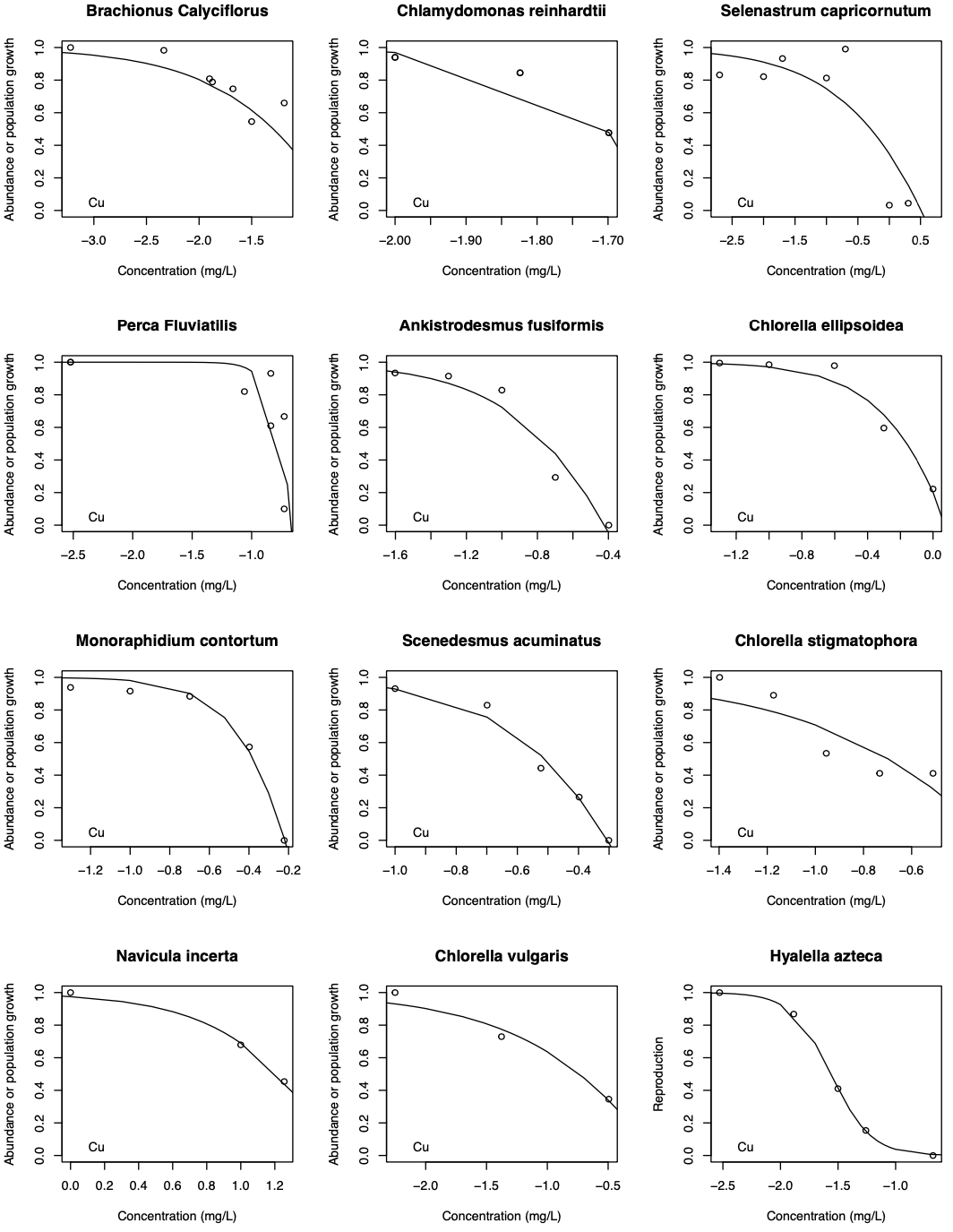
**

**Figure S3:** First part of the species-specific exposure-response data and curves obtained for Copper (Cu).

**
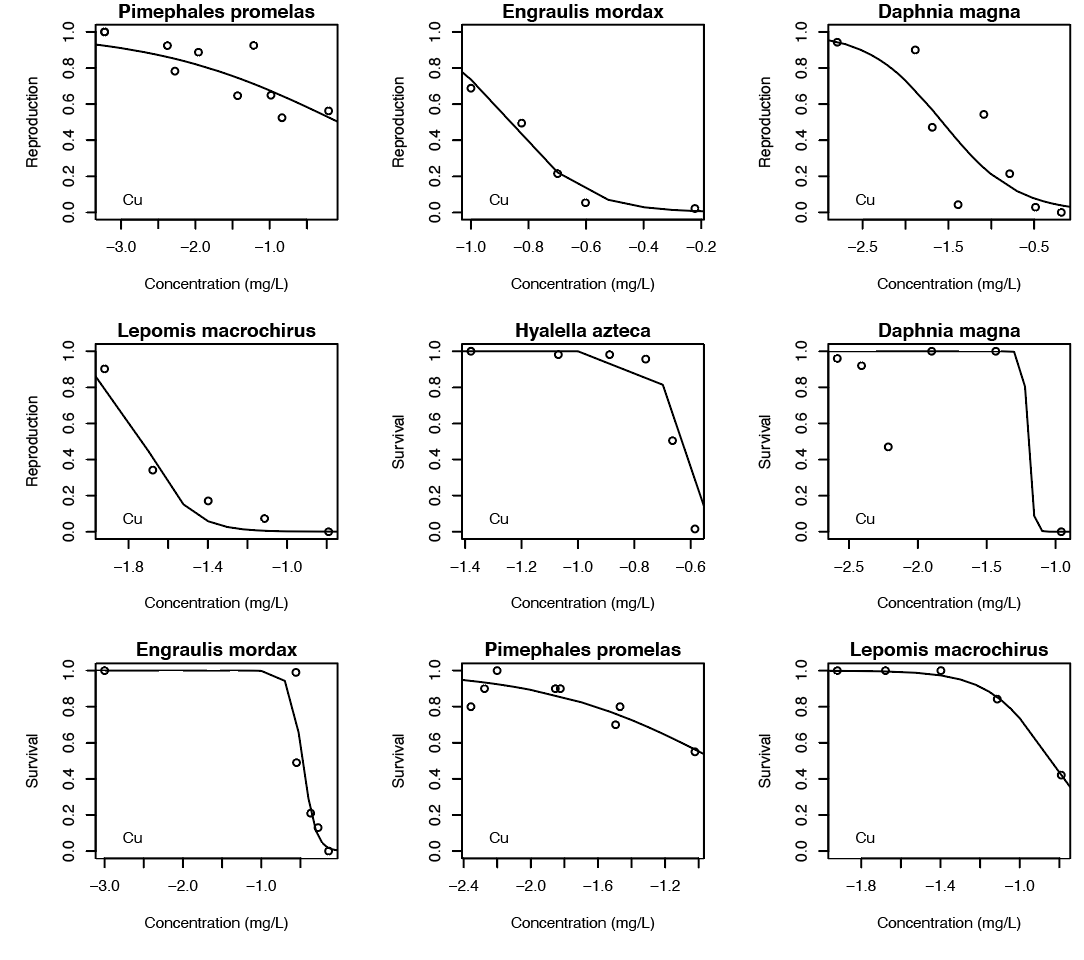
**

**Figure S4:** Second part of the species-specific exposure-response data and curves obtained for Copper (Cu).


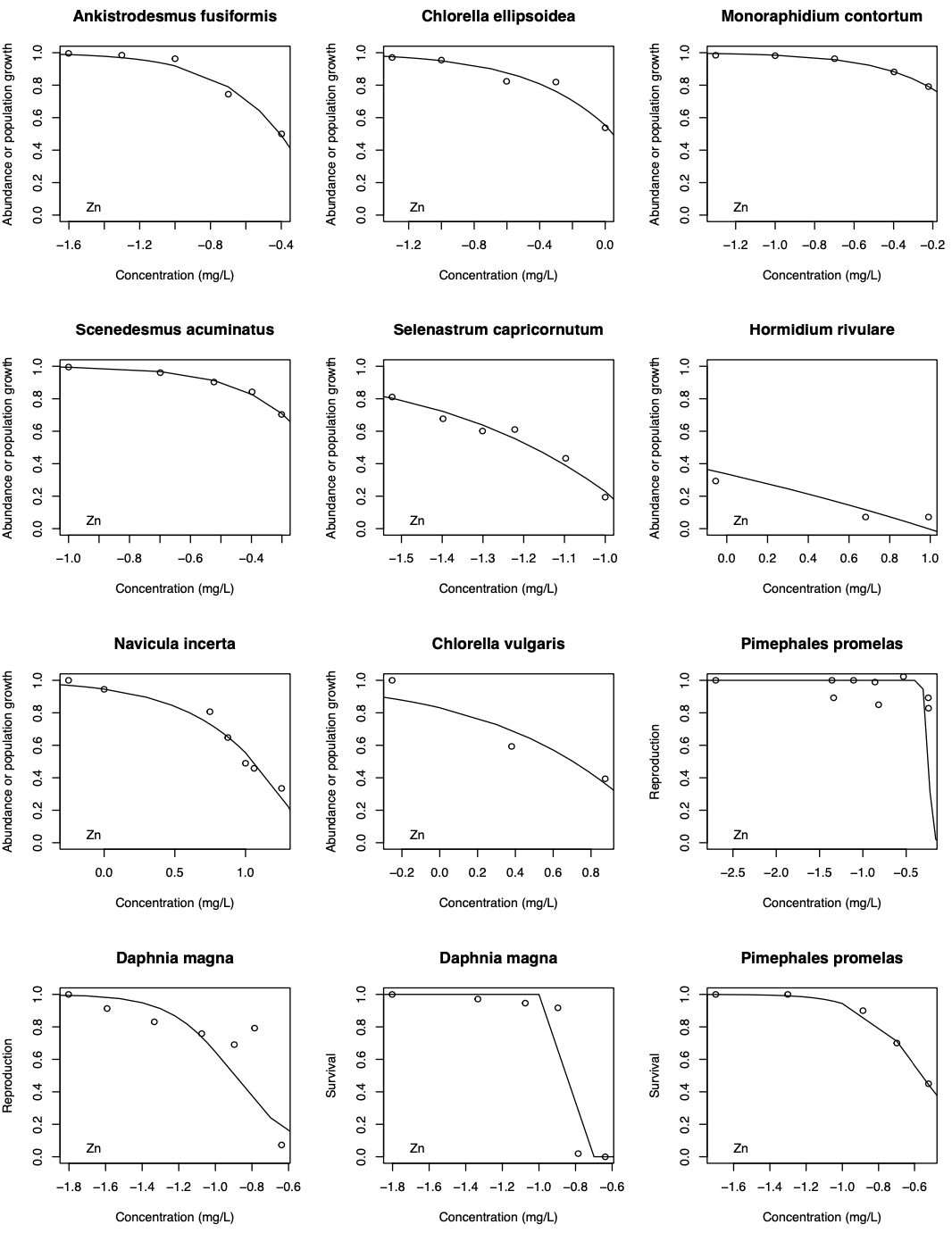


**Figure S5:** All species-specific exposure-response data and curves obtained for Zinc (Zn).

### Section 2. Population growth parameters

In order to calculate exposure-abundance relationships described by Equation 4 (see main text)$,$information about the species-specific undisturbed lifetime fecundity ($R_{0}$) is required. Savage et al. (2004) states that$r$ is inversely related to $Tg$, thus variation in$R_{0}$ between species is relatively low and can even be considered constant. Blueweiss et al. (1978) and Hendriks (2007) indicate the existence of strong correlations between body weight and intrinsic rate of increase, *r* (allometric relationships). Additionally, both papers provide insight into the relation between body weight and generation time $(Tg$). According to the regression results by Hendriks (2007), the intercepts for intrinsic rate of increase ($r)$ for both heterotherms and homeotherms show little variation. Under the assumption that $R_{0}$ can be realistically estimated via Equation 3 (see main text), which can be rewritten as $R_{0}= e^{r * Tg}$, and that the provided regression coefficients (Blueweiss et al. 1978; Hendriks 2007) hold for a range of species under optimal circumstances, a relationship can be obtained between body weight and lifetime fecundity, $R_{0}$ (see Table S4). Besides expressing the intrinsic rate of increase ($r),$ Hendriks (2007) also defines an estimated of the intrinsic rate of increase by utilizing the rate constant for production ($k_{p}$) and $R_{0}$ (Equation S1), assuming that generation time $(Tg$) is closely related to the juvenile period$(Tm$).

$$r\approx M\ln\left( R_{0} \right)k_{p} \left( Equation S1 \right)$$

The factor *M* in Equation S1 is a constant ranging from 0.5 to 1.5, depending on egg to adult mass and juvenile to adult mass ratios. By rewriting Equation S6 the allometric relationships in $r$ and $k_{p}$ can be used to estimate $R_{0}$:

$$R_{0}= e^{\frac{r}{M\cdot k_{p}}} \left( Equation S2 \right)$$

By making use of Equation S2 and the corresponding allometric relationships, Hendriks^15^ estimates the maximum time fecundity ($R_{0}$) to be about e^1.5^ = 4.5 to e^4^ = 55 for both heterotherms and homeotherms (see Table S4).

**Section 3. Supplementary information results**

This section includes the additional results not presented in the main text. A comparison between the log-logistic fit on the MSAR and the log-logistic EC_10_-based SSD curve (computed reproduction data extracted from our data set) is presented in Fig. S6. The expected total range in *R_0_* derived from allometric relationships presented in Blueweiss et al. (1978) and Hendriks (2007) is quantified in Table S4.

**
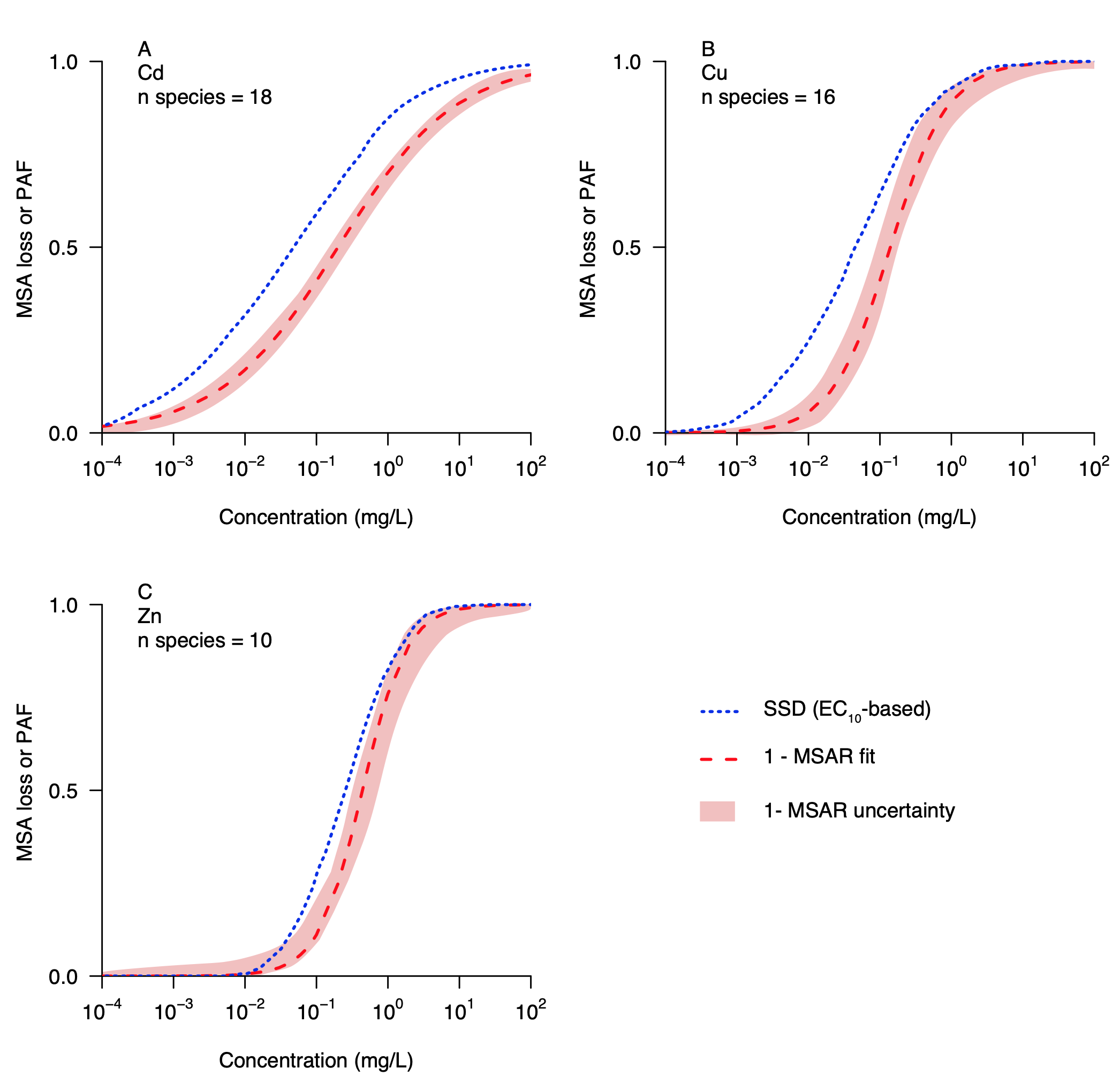
**

**Figure S6:** MSA loss and the potentially affected fraction of species (PAF) for A: cadmium (Cd), B: copper (Cu) and C: zinc (Zn). The red dashed lines indicate relationship between the MSA loss determined by 1 – MSAR (log-logistic fit) and the chemical concentration. Red areas surrounding the MSAR curves show the 95% confidence intervals of the simulated data (1,000 iterations). Dotted blue lines depict the (log-logistic fit) SSDs fitted on EC_10_ data for the most sensitive endpoint (i.e., reproduction), illustrating the relationship between the PAF and the chemical concentration.

**Table S4:** Range in $R_{0}$ explained by considering allometric relationships relating body weight to intrinsic rate of increase ($r)$, generation time $(Tg$) and rate constant for production ($k_{p}$)

| **Reference to allometric parameters used** | **Equation used for estimating** $\boldsymbol{R}_{\boldsymbol{0}}$ **range** | **Estimated maximum** $\boldsymbol{R}_{\boldsymbol{0}}$ **range** |
| --- | --- | --- |
| Blueweiss et al. 1978 ($r$, $Tg$) | $R_{0}= e^{r \cdot Tg}$ | 3.5-4.9 |
| Hendriks 2007 ($r$, $Tg$) | $R_{0}= e^{r \cdot Tg}$ | 2-11.5 |
| Hendriks 2007 ($r$, $Tg$, M) | $R_{0}= e^{\frac{r}{M\cdot k_{p}}}$ | 4.5-55 |

**Section 4: Simplifying the MSAR**

The following demonstrates why the simplified MSAR based on a single endpoint and without intra-species variation mostly follows the cumulative distribution of the EC50-values and is therefore highly similar to an EC_10_-based SSD. Equation 4 can be simplified by removing one of the two endpoints (e.g. survival) and excluding intra-species variation ($\beta$ = infinite), which results in Equation S3.

| $\frac{K\left( c \right)}{K\left( 0 \right)}= \frac{-ln \left( 1+\left( \frac{C}{EC_{50}} \right)^{inf} \right)}{ln(R_{0}(0))}+1$ (Equation S3) |  |
| --- | --- |

The resulting abundance curves can only equal 1 (when *EC50 > C*), *-ln(2) / ln(R_0_*$(0)$*)* +1 (at *C = EC_50_*) and 0 (when *EC50 < C*). Next, the MSAR can be computed from Equation 5 (in the main text), which averages the abundances obtained over a gradient of concentrations. In order for the MSAR to follow the cumulative distribution of the EC_50_-values, $K(c)/K\left( 0 \right)$ ratios need to 0.5 at *C = EC_50_*. This is true for when R_0_$(0)$ = 4 (see Equation S4).

$\frac{-ln \left( 1+\left( 1 \right)^{inf} \right)}{ln(4)}+1=0.5$ (Equation S4)

Considering the small range of R_0_$(0)$ values obtained from the allometric relationships and the fact that they are on average around 4, the values resulting from Equation S4 will not deviate far from 0.5 at *C = EC_50_*.

**References**

Azevedo MM. Cássio F. 2010. Effects of metals on growth and sporulation of aquatic fungi. *Drug Chem Toxicol*. 33(3):269-78.

Barata C. Donald JB. 2000. Determining the ecotoxicological mode of action of chemicals from measurements made on individuals: results from instar-based tests with Daphnia magna Straus. *Aquat. Toxicol..* 48.2-3:195-209.

Bartlett L, Rabe FW, Funk WH. Effects of copper, zinc and cadmium on Selanastrum capricornutum. Water Research. 1974 Mar 1;8(3):179-85.

Bengtsson BE. Bergström B. A flowthrough fecundity test with Nitocra spinipes (harpacticoidea crustacea) for aquatic toxicity. *Ecotoxicol. Environ. Saf.* 1987 14(3):260-8.

Bengtsson BE. Effect of zinc on growth of the minnow Phoxinus phoxinus. Oikos. 1974 Jan 1:370-3.

Benoit DA. Holcombe GW. 1978. Toxic effects of zinc on fathead minnows Pimephales promelas in soft water. *J. Fish Biol.* 13(6), 701-708.

Benoit DA. Chronic effects of copper on survival, growth, and reproduction of the bluegill (Lepomis macrochirus) Transactions of the American Fisheries Society. 1975 104(2): 353-358.

Blueweiss L. Fox H. Kudzma V. Nakashima D. Peters R. Sams S. 1978. Relationships between body size and some life history parameters. *Oecologia* 37(2):257-272.

Borgmann U. Norwood WP. Clarke C. 1993. Accumulation, regulation and toxicity of copper, zinc, lead and mercury in Hyalella azteca. *Hydrobiologia*, 259(2), 79-89.

Brinkman S. Vieira NM. 2008. Water pollution studies. *Colorado Division of Wildlife, Fish Research Section*.

Brown V, Shurben D, Miller W, Crane M. Cadmium toxicity to rainbow trout Oncorhynchus mykiss Walbaum and brown trout Salmo trutta L. over extended exposure periods. Ecotoxicology and environmental safety. 1994 Oct 1;29(1):38-46.

Brungs WA. Geckler JR. Gast M. 1976. Acute and chronic toxicity of copper to the fathead minnow in a surface water of variable quality. *Water Res.* 10(1), 37-43.

Collvin L. The effects of copper on maximum respiration rate and growth rate of perch, Perca fluviatilis L. Water research. 1984 Jan 1;18(2):139-44.

Dave G. 1984. Effects of copper on growth, reproduction, survival and haemoglobin in Daphnia magna. *Comp. Biochem. Physiol. C.* 78(2), 439-443.

ECB. 2007. Risk assessment cadmium metal, cadmium oxide. Institute for Health and Consumer Protection, Toxicology and Chemical Substances, European Chemicals Bureau, European Commission, Joint Research Centre, Ispra, Italy.

ECB. 2008. European Union risk assessment report on zinc, Part 1 -environment. European Chemicals Bureau, EUR 24587. 710 pp. https://doi.org/10.2788/40041.

ECHA. 2007. Voluntary risk assessment reports - Copper and Copper Compounds. European Chemicals Agency. <http://echa.europa.eu/web/guest/copper-voluntary-risk-assessment-reports>.

Hargreaves JW. Whitton BA. 1976. Effect of pH on tolerance of Hormidium rivulare to zinc and copper. *Oecologia.* 26(3), 235-243.

Harrison FL. Knezovich JP. Rice DW. 1984. The toxicity of copper to the adult and early life stages of the freshwater clam, Corbicula manilensis. *Arch. Environ. Contam. Toxicol.* 13(1), 85-92.

Hatakeyama S. Yasuno M. 1981. Effects of cadmium on the periodicity of parturition and brood size of Moina macrocopa (Cladocera). *Environ. Pollut. Series, Ecol. and Biol.* 26(2):111-20.

Hendriks AJ. 2007. The power of size: a meta-analysis reveals consistency of allometric regressions. *Ecol. Model.*205(1-2):196-208.

van der Heever JA. Grobbelaar JU. 1998. In VivoChlorophyll A Fluorescence of Selenastrum capricornutum as a Screening Bioassay in Toxicity Studies. *Arch. Environ. Contam. Toxicol.* 35(2), 281-286.

Indeherberg MB. Van Straalen NM. Schockaert ER. 1999. Combining life-history and toxicokinetic parameters to interpret differences in sensitivity to cadmium between populations of Polycelis tenuis (Platyhelminthes). *Ecotoxicol. Environ. Saf.* 44(1):1-1.

van Leeuwen CJ. Luttmer WJ. Griffioen PS. 1985. The use of cohorts and populations in chronic toxicity studies with Daphnia magna: a cadmium example. *Ecotoxicol. Environ. Saf.* 9(1):26-39.

Les A. Walker RW. 1984. Toxicity and binding of copper, zinc, and cadmium by the blue-green alga, Chroococcus paris. *Water Air Soil Pollut.* 23(2), 129-139.

Ngo HT. Gerstmann S. Frank H. 2009. Toxicity of cadmium to the green alga Parachlorella kessleri: Producing Cd-loaded algae for feeding experiments. *Toxicol Environ. Chem*. 91(2):279-88.

Magdaleno A, Vélez CG, Wenzel MT, Tell G. Effects of cadmium, copper and zinc on growth of four isolated algae from a highly polluted Argentina river. Bulletin of environmental contamination and toxicology. 2014 Feb 1;92(2):202-7.

McKim JM, Benoit DA. Effects of long-term exposures to copper on survival, growth, and reproduction of brook trout (Salvelinus fontinalis). Journal of the Fisheries Board of Canada. 1971 May 1;28(5):655-62

Mount DI. Chronic toxicity of copper to fathead minnows (Pimephales promelas, Rafinesque). Water Research. 1968 Mar 1;2(3):215-23.

Münzinger A. Monicelli F. 1991. A comparison of the sensitivity of three Daphnia magna populations under chronic heavy metal stress. *Ecotoxicol. Environ. Saf.* 22(1), 24-31.

Othman MS. Pascoe D. 2002. Reduced recruitment in Hyalella azteca (Saussure, 1858) exposed to copper. *Ecotoxicol. Environ. Saf*, 53(1), 59-64.

Pérez E, Hoang TC. Chronic toxicity of binary‐metal mixtures of cadmium and zinc to Daphnia magna. Environmental toxicology and chemistry. 2017 Oct;36(10):2739-49.

Pickering Q, Brungs W, Gast M. Effect of exposure time and copper concentration on reproduction of the fathead minnow (Pimephales promelas). Water Research. 1977 Jan 1;11(12):1079-83.

Prasad PD, Prasa PD. Effect of cadmium, lead and nickel on three freshwater green algae. Water, Air, and Soil Pollution. 1982 Apr 1;17(3):263-8.

Rachlin JW, Jensen TE, Warkentine B. The growth response of the diatom Navicula incerta to selected concentrations of the metals: cadmium, copper, lead and zinc. Bulletin of the Torrey Botanical Club. 1983 Apr 1:217-23.

Rice DW, Harrison FL. Copper sensitivity of the Northern anchovy, Engraulis mordax, during its early life history. California Univ.; 1978.

Rosko JJ. Rachlin JW. 1977. The effect of cadium, copper, mercury, zinc and lead on cell division, growth, and chlorophyll a content of the chlorophyte Chlorella vulgaris. Bull. Torrey Bot. Club. 226-233.

Savage VM. Gillooly JF. Brown JH. West GB. Charnov EL. 2004. Effects of body size and temperature on population growth. *Am. Nat.*. 163(3), 429-441.

de Schamphelaere KA. Janssen CR. 2010. Cross-phylum extrapolation of the Daphnia magna chronic biotic ligand model for zinc to the snail Lymnaea stagnalis and the rotifer Brachionus calyciflorus. *Sci. Total Environ.* 408(22), 5414-5422.

Sieratowicz A. Stange D. Schulte-Oehlmann U. Oehlmann J. 2011. Reproductive toxicity of bisphenol A and cadmium in Potamopyrgus antipodarum and modulation of bisphenol A effects by different test temperature. *Environ. pollut.* 159(10):2766-74.

Snell TW, Moffat BD. A 2‐d life cycle test with the rotifer Brachionus calyciflorus. Environmental Toxicology and Chemistry: An International Journal. 1992 Sep;11(9):1249-57.

Suedel BC, Deaver E, Rodgers JH. Experimental factors that may affect toxicity of aqueous and sediment-bound copper to freshwater organisms. Archives of Environmental Contamination and Toxicology. 1996 Jan 1;30(1):40-6.

Winner RW, Owen HA. Seasonal variability in the sensitivity of freshwater phytoplankton communities to a chronic copper stress. Aquatic toxicology. 1991 Apr 1;19(2):73-88.
